# A HfO_2_/SiTe Based Dual-Layer Selector Device with Minor Threshold Voltage Variation

**DOI:** 10.3390/nano9030408

**Published:** 2019-03-11

**Authors:** Bing Song, Rongrong Cao, Hui Xu, Sen Liu, Haijun Liu, Qingjiang Li

**Affiliations:** 1 College of Electronic Science, National University of Defense Technology, Changsha 410073, China; songbing@nudt.edu.cn (B.S.); xuhui@nudt.edu.cn (H.X.); liusen@nudt.edu.cn (S.L.); liuhaijun@nudt.edu.cn (H.L.); 2 Key Laboratory of Microelectronic Devices & Integrated Technology, Institute of Microelectronics, Chinese Academy of Science, Beijing 100029, China; caorongrong@ime.ac.cn

**Keywords:** chalcogenide, memristor, resistive random access memory, dual-layer, threshold switching selector

## Abstract

Volatile programmable metallization cell is a promising threshold switching selector with excellent characteristics and simple structures. However, the large variation of threshold voltage is a major problem for practical application. In this work, we propose a dual-layer structure to increase selectivity and improve the threshold voltage variation. Compared to single-layer devices, this dual-layer device exhibits higher selectivity (>10^7^) and better threshold voltage uniformity with less than 5% fluctuation during 200 DC switching. The improvement is attributed to good control on the location of the filament formation and rupture after introducing a HfO_2_ layer. It is deduced that a major factor consists of the difference of Ag ions mobility between SiTe and HfO_2_ due to the grain boundary quantity.

## 1. Introduction

With the end of Moore’s Law in sight, the memristor is emerging as a promising technology that could fill the power vacuum in semiconductor industry [1]. Three applications may significantly benefit from memristor developments: On-chip memory and storage [2,3], biologically inspired computing [4,5] and in-memory computing [6,7]. In order to exploit the full-scale strength of the memristor in crossbar array, a selector is necessary to suppress leakage current which influences array performance and power consumption [8,9]. Among various concepts of selector device, programmable metallization cell (PMC) is one of the most promising candidates; it exhibits excellent threshold switching characteristics and simple material stacks [10,11,12]. However, the issue of variation on threshold switching voltage becomes a major problem in one-selector-one-resistor (1S1R) array integration and operation [9,13,14]. In our previous work, the SiTe based PMC selector was improved by annealing but still with large variation [15]. SiTe is selected because it is promising for threshold switching owing to rigid structure and stability. A rigid structure helps avert strong metal filament while assuring volatile switching instead of memory.

According to published research findings on CBRAM (conductive bridge random access memory) [16,17,18,19,20], bilayer devices exhibit better memory characteristics such as stable switching voltages and on/off ratios, compared to single layer devices by controlling conductive filament size and position. Inspired by this view, we proposed a dual-layer PMC selector based on SiTe and HfO_2_. The results indicate that the threshold switching characteristics of the dual-layer devices can be improved with high selectivity of 10^7^ and highly uniform threshold voltage. The possible mechanism for improvement of the volatile switching characteristics in the dual-layer device is deduced in detail.

## 2. Materials and Methods 

**Figure 1:**
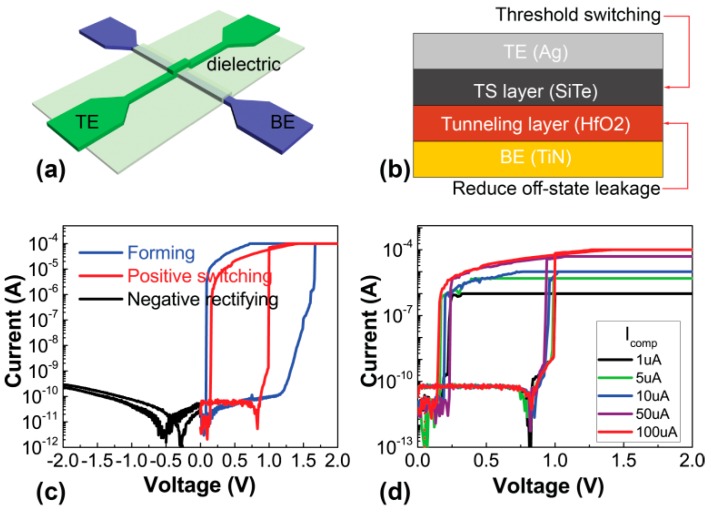
(**a**) Schematics of crosspoint device structure (**b**) Schematics of the asymmetric device composition. (**c**) DC I-V characteristic of the dual-layer selector in various biases. (**d**) Threshold switching behaviors of the dual-layer device at different compliance currents.

The SiTe/HfO_2_ dual-layer selector devices were fabricated with a crossbar structure on a p-type Si wafer with 200 nm thermal oxide. The device areas range from 2 × 2 μm^2^ to 50 × 50 μm^2^, as illustrated in Figure 1a,b. The device size with micrometer scale is not state of the art. We designed this dual-layer device to verify the validity of variation mitigation. Owing to a local conductive mechanism with conductive filaments, we suppose that the electrical characteristics will be similar to the device with micrometer size but the switching current may be reduced. [21,22] Firstly, photo-lithography was implemented to pattern the bottom electrode (BE) and 40 nm TiN was deposited with ion beam sputtering. Consequently, 2 nm thick HfO_2_ was deposited by atomic layer deposition (ALD) as a tunneling layer to increase selectivity [23]. Then, 50 nm SiTe was deposited to the full wafer with magnetron sputtering method at room temperature. The wafer was annealed under vacuum at 300 °C for 30 s to reduce traps. Finally, 40 nm Ag (TE) was patterned and deposited with ion beam sputtering to form an Ag/SiTe/HfO_2_/TiN PMC device.

The measurements were carried out with a standard semiconductor parameter analyzer (Keithley 4200 SCS, Beaverton, OR, USA). The TE (Ag) was biased and the BE (TiN) was grounded during the measurements. Typical threshold switching is realized under positive stimulus while a rectifying effect is available under negative stimulus after a forming operation, as in Figure 1c. Figure 1d shows asymmetric threshold switching behaviors of the dual-layer device at different compliance currents without obvious degradation up to 100 μA.

## 3. Results

**Figure 2:**
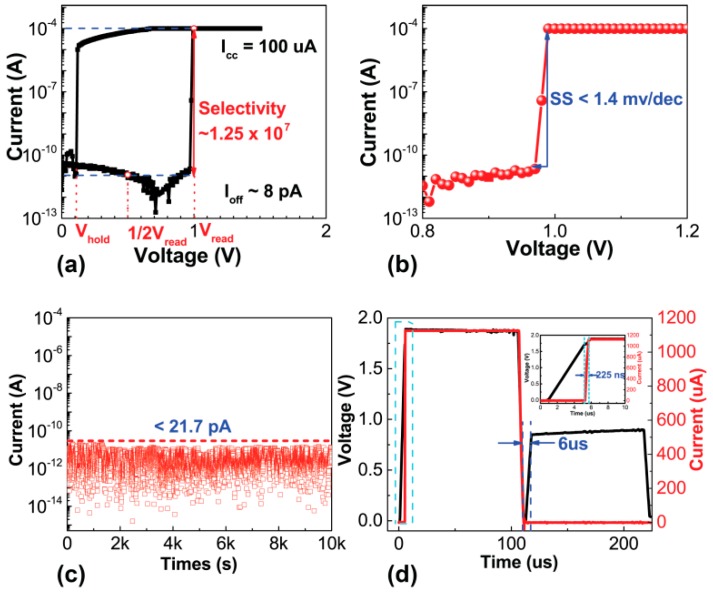
(**a**) Typical DC I-V curve of the dual-layer selector. (**b**) Zoomed-in plot of (a) shows extremely steep switching slope less than 1.4 mV/dec. (**c**) DC stress test of the device without obvious degradation of the OFF state at room temperature. (**d**) Pulse measurements of the device. The device can be turned on within 225 ns (inset zoomed-in figure), and relaxed within 6 µs at 2 V for 100 µs operating pulse.

Figure 2a demonstrates I-V characteristics of the as-fabricated selector at 100 μA I_comp_ while voltage sweeps forward and backward, respectively. The mechanism of volatile and non-volatile switching in PMC device are the same. When the filament of active atoms connects to the electrodes under bias, the device turns to a low-resistance state. However, the difference is that the volatile switching device returns back to high-resistance state after the bias is removed while the non-volatile switching device remains low resistance. By controlling the filament thickness, volatile and non-volatile switching can be achieved in the same device. In this case of selector, volatile switching rather than non-volatile switching is attained with appropriate compliance current. When the applied voltage sweeps from 0 V to 1.5 V, the device is initially at high-resistance state (OFF) and then abruptly turns to low resistance state (ON) at ~1.0 V threshold voltage (V_th_), with current immediately reaching I_comp_. When the voltage sweeps back, the device maintains the ON state until holding voltage (V_hold_) of 0.1 V and abruptly turns to the OFF state. The OFF state current is around 8 pA, which is limited by the accuracy of test instrument. Therefore, the selectivity is 1.25 × 10^7^, which can effectively prevent leakage current in the crossbar array. Figure 2b shows the zoomed-in plot of Figure 2a, in which the device exhibits extremely steep switching slope lower than 1.4 mV/dec. DC stress test of the selector shows that the current is less than 20 pA and there is no obvious degradation of the device OFF state with 0.5 V bias (V_read_/2) for 10^4^ s at room temperature (Figure 2c). Figure 2d shows the ON-OFF switching by pulse measurements. The device can be turned on within 225 ns at 2 V with 100 μs operating pulse (inset zoomed-in figure), and quickly recovers to the OFF state once the voltage is removed, with a recovery time lower than 6 μs. The 100 μs width is utilized to guarantee successful operation. The as-fabricated devices manifest excellent selectivity and fast switching speed, which benefit the large memristor crossbar array to suppress the leakage current.

**Figure 3:**
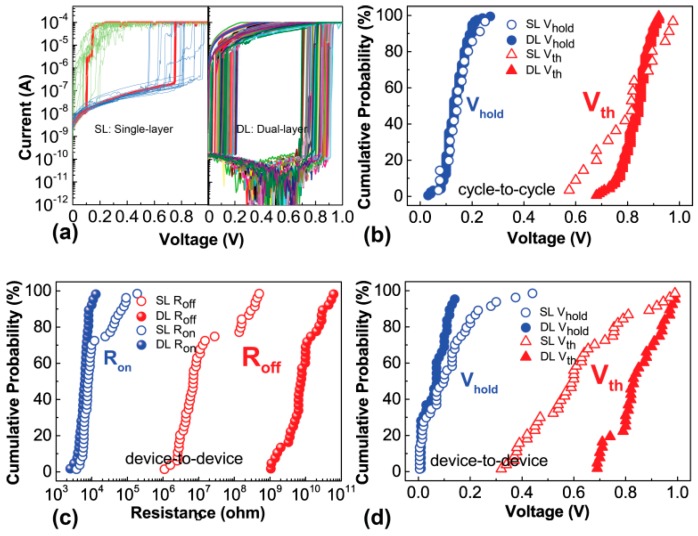
(**a**) The DC cycling test shows stably repeatable volatile threshold switching. Inset shows large variation of threshold voltage of the single-layer device. (**b**) Cycle-to-cycle variety of threshold voltage and holding voltage. (**c**) Device-to-device variety of OFF and ON resistance. (**d**) Device-to-device variety of threshold voltage and holding voltage.

The switching stability and uniformity of the dual-layer devices are further studied and compared to single-layer devices. The cycle-to-cycle uniformity is demonstrated in Figure 3a,b with 200 consecutive DC sweeps. In Figure 3a, the dual-layer selector shows stable volatile threshold switching characteristic without obvious degradation of OFF state and selectivity. The inset is the DC cycles of the single-layer device with large variation of threshold voltage. Simultaneously, the variation of threshold voltage and holding voltage are shown in cumulative probability (Figure 3b). It indicates tight distribution of voltages in dual-layer device, which is beneficial for the array operation. Figure 3c,d shows the uniformity of resistance and voltage of the device-to-device from 30 dual-layer devices and single-layer devices. The results show that the distribution of ON state is tighter than OFF state which surpasses 1 GΩ in dual-layer devices [24]. In this case of study, the origin of variance is dependent upon randomly oriented local disorders within the active layer that have a substantial impact on the overall state variance, particularly for high-resistance state. Specifically, there is no continuous conductive filament in the high-resistance state and randomly local disorders in the active layer seriously impact the current. However, there is continuous filament in the low-resistance state and local disorders will not play a big role. As a result, the variance of ON state is relatively lower than the OFF state. In addition, the threshold voltage spans from 0.7 V to 1.0 V and the distribution of hold voltage is tighter in dual-layer devices. From the aforementioned results, stability and uniformity can be greatly improved by inserting HfO_2_ in the dual-layer device and it helps the integration of selector and memristor.

**Figure 4:**
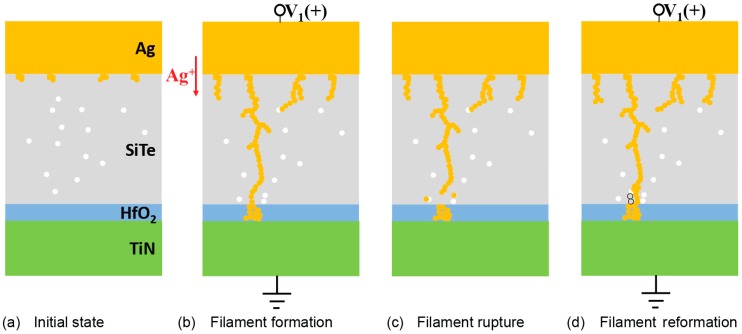
Schematic representation of controlled filament formation and rupture in dual-layer devices. Yellow dot represents Ag atoms; white dot represents defects. (**a**) The initial state is highly resistive. (**b**) Conductive filament randomly forms for the first time. (**c**) Filament ruptures in SiTe because of the interfacial energy and large mobility. (**d**) Filament reconnects near previous filament location.

The above results show dual-layer selector characteristics with stably repeatable threshold switching and less variation of threshold voltage by inserting the HfO_2_ layer. Nevertheless, the function of HfO_2_ is not the tunneling layer as designed because the holding voltage is near zero, as in Figure 2a. We suppose that the enhancement of threshold switching characteristics is realized by the precise control of the silver filament growth and the process is schematically explained in Figure 4 [16,17,18,19,20]. Firstly, there are few Ag atoms in the interface of the Ag electrode and the SiTe layer during the sputtering process and the initial state of the device is highly resistive without complete filament (Figure 4a). In Figure 4b, when applying positive voltage to the top electrode, the Ag atoms get oxidized and become Ag ions. Ag ions randomly drift from TE towards BE under an electric field and are reduced on the BE. When reduced Ag atoms increase and accumulate in the electrolyte, the filament is formed. If there is only a SiTe layer, many stochastic filaments form in the layer owing to large density of grain boundaries in amorphous chalcogenide [25,26,27,28]. Due to the randomness of local filament formation, the connecting filament in following successive cycles may be different, leading to large variation of threshold voltage and resistance [29]. When the HfO_2_ layer is inserted, Ag atoms must traverse the HfO_2_ layer before the filament is formed. However, the trap is rare in dense HfO_2_ with the ALD process which is realized by depositing the atoms layer by layer. As a result, large voltage is needed to move Ag ions through the HfO_2_ and atoms accumulate in the HfO_2_ until the filament forms. When the voltage is removed (as in Figure 4c), the thin Ag filament spontaneously ruptures because of the interfacial energy driven diffusion mechanism [12,30]. Specifically, when bias is removed, Ag atoms ionize due to the high surface energy associated with high chemical reactivity around the thin Ag filament. Thus, the filament is ionized to minimize the steric repulsion between the Ag atoms and the surrounding electrolyte. When the radius of the filament is decreased, the instability of the surface atoms is accelerated, the atomic combination of the filament is finally broken and the device returns back to OFF state. Since the Ag mobility in SiTe is larger than in HfO_2_ owing to the high grain boundary density, filament ruptures happen at the conjunction of SiTe and HfO_2_ [18]. The device returns to high-resistance state but some residual atoms are retained in the HfO_2_ layer. In the following cycle, the filament retained in the HfO_2_ layer increases the electric field and guides the filament connection in the neighborhood (shown in Figure 4d). In this way, the position of the filament can be confined at certain points, avoiding random formation and rupture under continuous cycles. Therefore, stable and uniform threshold switching is obtained with minor threshold voltage variation.

## 4. Conclusions

In conclusion, we demonstrated an excellent selector based on SiTe/HfO_2_ stack. Excellent selector properties including high selectivity (1.25 × 10^7^), high on-current drive (100 μA) and steep switching slope (<1.4 mV/dec) were achieved. Furthermore, the dual-layer device shows minor variation of threshold voltage and the deduced mechanism is because of the controlled filament location. Excellent volatile threshold switching and minor variation have potential application as a selector for large memristor crossbar arrays. The presented selector herein demonstrated unipolar threshold switching behavior which is only suitable for unipolar memristor. Bipolar threshold switching is our next-step research direction.

## References

[1] Zidan M.A., Strachan J.P., Lu W.D. (2018). The future of electronics based on memristive systems. Nat. Electron..

[2] Zaffora A., Cho D.Y., Lee K.S., Quarto F.D., Waser R., Santamaria M., Valov I. (2017). Electrochemical Tantalum Oxide for Resistive Switching Memories. Adv. Mater..

[3] Mao M., Yu S., Chakrabarti C. (2018). Design and Analysis of Energy-Efficient and Reliable 3-D ReRAM Cross-Point Array System. IEEE Trans. VLSI Syst..

[4] Burr G.W., Shelby R.M., Sebastian A., Kim S., Kim S., Sidler S. (2017). Neuromorphic computing using non-volatile memory. Adv. Phys. X.

[5] Choi S., Tan S.H., Li Z., Kim Y., Choi C., Chen P.Y., Yeon H., Yu S., Kim J. (2018). SiGe epitaxial memory for neuromorphic computing with reproducible high performance based on engineered dislocations. Nat. Mater..

[6] Ielmini D., Wong H.S.P. (2018). In-memory computing with resistive switching devices. Nat. Electron..

[7] Chi P., Li S., Xu C., Zhang T., Zhao J., Liu Y., Wang Y., Xie Y. PRIME: A Novel Processing-in-Memory Architecture for Neural Network Computation in ReRAM-Based Main Memory. Proceedings of the 2016 ACM/IEEE 43rd Annual International Symposium on Computer Architecture (ISCA).

[8] Burr G.W., Shenoy R.S., Virwani K., Narayanan P., Padilla A., Kurdi B., Hwang H. (2014). Access devices for 3D crosspoint memory. J. Vac. Sci. Technol. B.

[9] Song B., Xu H., Liu H., Li Q. (2017). Impact of threshold voltage variation on 1S1R crossbar array with threshold switching selectors. Appl. Phys. A-Mater..

[10] Zhao X., Ma J., Xiao X., Liu Q., Shao L., Chen D., Liu S., Niu J., Zhang X., Wang Y. (2018). Breaking the Current-Retention Dilemma in Cation-Based Resistive Switching Devices Utilizing Graphene with Controlled Defects. Adv. Mater..

[11] Yoo J., Park J., Song J., Lim S., Hwang H. (2018). Field-induced nucleation in threshold switching characteristics of electrochemical metallization devices. Appl. Phys. Lett..

[12] Lee T.H., Kang D.Y., Kim T.G. (2018). Ag:SiOxNy-Based Bilayer ReRAM Structure with Self-Limiting Bidirectional Threshold Switching Characteristics for Cross-Point Array Application. Appl. Phys. Lett. ACS Appl. Mater. Interface.

[13] Chen A., Lin M.R. Variability of resistive switching memories and its impact on crossbar array performance. Proceedings of the 2011 IEEE International Reliability Physics Symposium.

[14] Zhang L., Cosemans S., Wouters D.J., Groeseneken G., Jurczak M., Govoreanu B. (2015). Cell Variability Impact on the One-Selector One-Resistor Cross-Point Array Performance. IEEE Trans. Electron Dev..

[15] Song B., Xu H., Liu S., Liu H., Li Q. (2018). Threshold Switching Behavior of Ag-SiTe-Based Selector Device and Annealing Effect on its Characteristics. IEEE J. Electron Devi..

[16] Kumar D., Aluguri R., Tsenga U.C.Y. (2017). Enhancement of resistive switching properties in nitride based CBRAM device by inserting an Al2O3 thin layer. Appl. Phys. Lett..

[17] Kumar D., Aluguri R., Tsenga U.C.Y. (2018). Role of Al2O3 thin layer on improving the resistive switching properties of Ta5Si3-based conductive bridge random accesses memory device. Jpn. J. Appl. Phys..

[18] Lv H., Wan H., Tang T. (2010). Improvement of Resistive Switching Uniformity by Introducing a Thin GST Interface Layer. IEEE Electron Dev. Lett..

[19] Kim D.C., Seo S., Ahn S.E., Suh D.-S., Lee M.J., Park B.-H., Yoo I.K., Baek I.G., Kim H.-J., Yim E.K. (2006). Improvement of resistive memory switching in NiO using IrO_2_. Appl. Phys. Lett..

[20] Ogimoto Y., Tamai Y., Kawasaki M., Tokura Y. (2007). Resistance switching memory device with a nanoscale confined current path. Appl. Phys. Lett..

[21] Lim S., Yoo J., Song J., Woo J., Park J., Hwang H. Excellent threshold switching device (IOFF ~1 pA) with atom-scale metal filament for steep slope (<5 mV/dec), ultra-low voltage (VDD = 0.25 V) FET applications. Proceedings of the 2016 IEEE International Electron Devices Meeting (IEDM).

[22] Shukla N., Grisafe B., Ghosh R.K., Jao N., Aziz A., Frougier J., Jerry M., Sonde S., Rouvimov S., Orlova T. Ag/HfO_2_ based Threshold Switch with Extreme Non-Linearity for Unipolar Cross-Point Memory and Steep-slope Phase-FETs. Proceedings of the 2016 IEEE International Electron Devices Meeting (IEDM).

[23] Luo Q., Xu X., Liu H., Lv H., Gong T., Long S., Liu Q., Sun H., Banerjee W., Li L. Cu BEOL Compatible Selector with High Selectivity Extremely Low Off-current and High Endurance. Proceedings of the 2015 IEEE International Electron Devices Meeting (IEDM).

[24] Salaoru I., Khiat A., Li Q., Berdan R., Papavassiliou C., Prodromakis T. (2014). Origin of the OFF state variability in ReRAM cells. J. Phys. D. Appl. Phys..

[25] Park G., Li X., Kim D., Jung R., Lee M., Seo S. (2007). Observation of electric-field induced Ni filament channels in polycrystalline NiOx film. Appl. Phys. Lett..

[26] Yang Y., Gao P., Li L., Pan X., Tappertzhofen S., Choi S.H., Waser R., Valov I., Lu W.D. (2014). Electrochemical dynamics of nanoscale metallic inclusions in dielectrics. Nat. Commun..

[27] Celano U., Giammaria G., Belmonte A., Jurczak M., Vandervorst W. (2016). Nanoscopic structural rearrangements of the Cu-filament in conductive-bridge memories. Nanoscale.

[28] Celano U., Goux L., Belmonte A., Opsomer K., Franquet A., Schulze A., Detavernier C., Richard O., Bender H., Jurczak M. (2014). Three-Dimensional Observation of the Conductive Filament in Nanoscaled Resistive Memory Devices. Nano Lett..

[29] Lv H.B., Yin M., Zhou P., Tang T.A., Chen B.A., Bao A., Chi M.H., Lin Y.Y. Improvement of endurance and switching stability of forming-free CuxO RRAM. Proceedings of the 23nd IEEE NVSMW, ICMTD.

[30] Sonde S., Chakrabarti B., Liu Y., Sasikumar K., Lin J., Stan L., Divan R., Ocola L.E., Rosenmann D., Choudhury P. (2018). Silicon compatible Sn-based resistive switching memory. Nanoscale.

